# Triglyceride/High-Density Lipoprotein Cholesterol (TG/HDL) Ratio May Predict Gestational Diabetes and Worse Cardiometabolic Profile

**DOI:** 10.7759/cureus.85505

**Published:** 2025-06-07

**Authors:** Gabriel G Souza Santos, Filipe D Souza, Micaela F Montero, Martha C Jordão, Maria Carolina M Oliveira, Carolina C Janovsky, Rosiane Mattar, Sergio A Dib, Patricia M Dualib, Bianca de Almeida-Pititto

**Affiliations:** 1 Department of Medicine, Universidade Federal de São Paulo, São Paulo, BRA; 2 Department of Endocrinology, Universidade Federal de São Paulo, São Paulo, BRA; 3 Department of Obstetrics and Gynecology, Universidade Federal de São Paulo, São Paulo, BRA; 4 Department of Preventive Medicine, Universidade Federal de São Paulo, São Paulo, BRA

**Keywords:** diagnosis of gdm, gestational diabetes mellitus (gdm), maternal cardiometabolic profile, obesity, tg/hdl ratio

## Abstract

Introduction

Current approaches to diagnosing and managing gestational diabetes mellitus (GDM) do not account for the diverse cardiometabolic (CM) profiles of pregnant women. The triglyceride/high-density lipoprotein cholesterol (TG/HDL) ratio is recognized as a useful marker of CM risk, but its significance during pregnancy remains unclear.

Objective

The objective of this study is to evaluate whether the TG/HDL ratio is associated with distinct CM profiles and the timing of GDM diagnosis.

Methods

This cross-sectional study included 651 women aged 18 years or older who received specialized antenatal care for GDM between January 2008 and November 2021. The TG/HDL ratio was assessed during the second or third trimester and categorized into tertiles: T1 (<2.22), T2 (2.22-3.37), and T3 (>3.37). Maternal characteristics and the timing of GDM diagnosis (first trimester vs. second/third trimester) were compared across tertiles.

Results

The mean TG/HDL ratio was 3.1 (SD 1.7). Increasing tertiles were associated with a progressive worsening of CM indicators. Women in higher tertiles had significantly higher pre-gestational BMI, fasting glucose, two-hour oral glucose tolerance test glucose, glycated hemoglobin, and triglyceride-glucose index values (all p < 0.05). The prevalence of obesity and GDM diagnosed in the second or third trimester also rose significantly across tertiles. A borderline association with hypertension was noted. Cesarean delivery rates were higher in the highest tertile, while other maternal-fetal complications showed no significant differences.

Conclusions

Elevated TG/HDL ratios are associated with a poorer CM profile and a higher likelihood of GDM diagnosis in the second or third trimester.

## Introduction

Gestational diabetes mellitus (GDM) is the most common metabolic disorder during pregnancy, affecting approximately 18% of pregnancies in Brazil and 14% worldwide [1,2]. It is associated with several risk factors, including overweight, obesity, advanced maternal age, and a prior history of GDM [3].

GDM has significant implications for both maternal and fetal health. It increases the risk of developing type 2 diabetes mellitus, hypertension, preeclampsia, cesarean delivery, and other uterine complications [4]. These outcomes are largely driven by hormonal and lipid imbalances, as well as the physiological increase in insulin resistance during pregnancy [5].

Despite its prevalence and impact, current diagnostic and treatment strategies for GDM do not account for variations in insulin resistance or the diverse CM profiles observed among pregnant women. These profiles are increasingly recognized as important for predicting maternal-fetal outcomes and guiding clinical management [6-8].

Outside of pregnancy, the triglyceride/high-density lipoprotein cholesterol (TG/HDL) ratio is a well-established marker of insulin resistance and impaired glucose tolerance. It also functions as an independent predictor of diabetes risk [9]. Beyond glycemic control, the TG/HDL ratio has been associated with cardiovascular outcomes, including atherosclerosis and ischemic stroke, even among individuals with low baseline risk [10,11]. These associations support its potential use as a practical tool for identifying individuals at elevated CM risk.

However, there is limited evidence on the performance of this marker during pregnancy. Investigating the TG/HDL ratio in this context may help define distinct GDM phenotypes and offer a low-cost, accessible, and reproducible option for assessing risk at any stage of gestation. Such stratification could support more tailored treatment approaches and ultimately improve maternal and fetal outcomes.

Against this backdrop, the present study aimed to evaluate the association between the TG/HDL ratio and CM profiles during pregnancy, as well as its relationship with the timing of GDM diagnosis.

## Materials and methods

Study design and participants

This cross-sectional study included data collected during the antenatal care of 651 women who attended the Diabetes and Pregnancy outpatient service at the Universidade Federal de São Paulo (UNIFESP) between January 2008 and November 2021. All participants were over 18 years old and diagnosed with GDM based on the diagnostic criteria established by the Brazilian Diabetes Society: fasting blood glucose >92 mg/dL and <126 mg/dL in the first trimester and/or altered results on the 75 g oral glucose tolerance test (OGTT) performed between 24 and 28 weeks of gestation (fasting glucose >92 mg/dL and/or one-hour >180 mg/dL and/or two-hour >153 mg/dL) [12]. All lipid measurements were performed after a minimum of 8 hours of fasting, following standard clinical protocols.

The study was approved by the Research Ethics Committee of the Paulista School of Medicine, São Paulo Hospital, UNIFESP (CAAE: 66952423.5.0000.5505). All participants provided written informed consent.

Variables of interest

Exposure

The primary exposure was the TG/HDL ratio measured during the second or third trimester of pregnancy. This variable was stratified into tertiles based on the distribution within the study sample (n = 651): T1 (< 2.22), T2 (2.22-3.37), and T3 (> 3.37). Due to the absence of established clinical cutoffs for TG/HDL during pregnancy, this stratification allowed for an exploratory analysis of dose-response trends and supports hypothesis generation for future research.

Outcomes

Primary outcome variables included indicators of the CM profile during pregnancy: nutritional status, lipid profile, glycemic measures, the triglyceride-glucose index (TyG index: log [(fasting triglycerides [mg/dL] × fasting glucose [mg/dL])/2]), timing of GDM diagnosis (first trimester vs. second/third trimester), and the presence of hypertension (preexisting or pregnancy induced).

Covariates

Covariates included age; pre-pregnancy BMI; gestational weight gain; blood pressure; fasting glucose; one-hour and two-hour OGTT values (if tested in the second or third trimester); lipid profile (total cholesterol and fractions and triglycerides); insulin use during pregnancy (percentage of users and dose per kg); personal medical history (diabetes-related and other chronic conditions); obstetric history; maternal complications (hypertension, preeclampsia, hypothyroidism, cesarean delivery, or prematurity); and fetal outcomes (malformations, NICU admission, hypoglycemia, hyperbilirubinemia, respiratory distress, neonatal death, low birth weight, or macrosomia).

Statistical analysis

Participants were grouped by TG/HDL tertiles, and outcomes and covariates were compared across these groups. The distribution and variance of continuous variables were assessed using descriptive plots and distributional statistics in IBM SPSS Statistics for Windows, Version 26.0 (Released 2018; IBM Corp., Armonk, NY, USA). Parametric tests (ANOVA) were used for normally distributed continuous variables, and the chi-square test was used for categorical variables.

Linear regression models were used to evaluate associations between TG/HDL (independent variable) and fasting glucose, glycated hemoglobin (HbA1c), two-hour OGTT glucose, and the TyG index (dependent variables). Model assumptions of normality and homogeneity of variances were verified.

Logistic regression models were used to examine associations between TG/HDL tertiles and binary outcomes, including hypertension, obesity, and timing of GDM diagnosis. Covariates were selected based on their known relevance to CM outcomes. Age and BMI were included due to their clinical significance and statistically significant differences across tertiles. Other variables, such as parity, gestational weight gain, and insulin use, did not differ significantly between groups and were excluded from the final models.

All statistical analyses were performed using IBM SPSS Statistics for Windows, Version 26.0. A p-value < 0.05 was considered statistically significant. Effect sizes were reported alongside significance values: eta-squared (η²) for ANOVA, ORs with 95% CIs for logistic regression, and unstandardized beta coefficients (β) with 95% CI for linear regression. Analyses were based on complete case data, and no imputation was performed for missing values.

## Results

The mean age of the 651 women included in the analysis was 33.8 years (SD 5.5). Most participants were either overweight or obese (77.8%) and had experienced two or more previous pregnancies (81.7%).

A progressive worsening of glycemic and lipid markers was observed across increasing TG/HDL tertiles. Women in the higher tertiles had significantly higher pre-gestational BMI, fasting glucose, two-hour OGTT glucose, HbA1c, total cholesterol, low-density lipoprotein (LDL) cholesterol, triglycerides, and TyG index values, along with lower HDL cholesterol levels. These trends indicate a progressively adverse CM profile with higher TG/HDL ratios.

Clinical outcomes followed a similar pattern. The prevalence of obesity, cesarean deliveries, and GDM diagnoses in the second or third trimester increased across tertiles. Notably, the cesarean delivery rate in the highest tertile (T3) was more than 30% higher than in the lowest tertile (T1). Hypertension showed a borderline association, with a trend toward higher prevalence in higher tertiles (p = 0.057). No significant differences were found for other maternal or fetal complications.

Comprehensive descriptive statistics are presented in Table 1.

**Table 1:** Characteristics of women with GDM and maternal-fetal outcomes according to TG/HDL tertiles during pregnancy ^*^ Obstetric personal history includes hypertension, preeclampsia, and hypothyroidism. ^**^ Maternal complications include hypertension, preeclampsia, and hypothyroidism. ^*** ^Neonatal complications include malformations, NICU admission, hypoglycemia, hyperbilirubinemia, respiratory distress, and neonatal death. Values are presented as mean (SD) or n (%). Continuous variables were analyzed using ANOVA with Bonferroni correction. If p < 0.05, pairwise differences are denoted as follows: “a” vs. Tertile 1; “b” vs. Tertile 2. Categorical variables were compared using the chi-square test. TG/HDL tertiles: tertile 1: <2.22; tertile 2: 2.22-3.37; tertile 3: >3.37 TyG index: log [(fasting triglycerides (mg/dL) × fasting glucose (mg/dL)) / 2] GDM, gestational diabetes mellitus; HbA1c, glycated hemoglobin; HDL, high-density lipoprotein; OGTT, oral glucose tolerance test; TG/HDL, triglyceride/high-density lipoprotein cholesterol; TyG, triglyceride-glucose index

Variable	Tertile 1 TG/HDL (<2.22)	Tertile 2 TG/HDL (2.22-3.37)	Tertile 3 TG/HDL (>3.37)	p	p for trend	Degrees of freedom	Effect size
Maternal characteristics							
Age (years)	33.8 (5.2)	34.1 (6.0)	33.7 (5.2)	0.703	0.801		
Pre-gestational BMI (kg/m²)	28.6 (5.7)	30.5 (6.6)^a^	30.6 (5.8)^a^	0.001	0.001		
BMI categories, n (%)				0.005	<0.001	4	15.094
Normal	65 (30.7)	40 (19.4)	33 (16.1)				
Overweight	66 (31.1)	68 (33)	68 (33.2)				
Obesity	81 (38.2)	98 (47.6)	104 (50.7)				
Parity, n (%)				0.154	0.087	4	6.67
0	58 (26.7)	60 (27.8)	54 (24.9)				
1	93 (42.9)	78 (36.1)	73 (33.6)				
2+	66 (30.4)	78 (36.1)	90 (41.5)				
Previous hypertension, n (%)	19 (9)	31 (14.9)	31 (15.3)	0.104	0.057	2	4.529
Hypothyroidism, n (%)	22 (10.5)	18 (8.7)	24 (11.9)	0.104	0.057	2	1.161
Smoking, n (%)	27 (12.9)	39 (18.8)	38 (18.7)	0.179	0.109	2	3.444
Time of diagnosis of GDM, n (%)				0.001	<0.001	2	14.453
First trimester	52 (24)	33 (15.2)	23 (10.6)				
Second/third trimester	165 (76)	184 (84.8)	194 (89.4)				
Glycemic and lipid markers							
Fasting glucose in the first trimester (mg/dl)	94.5 (7.2)	90.6 (9.0)^a^	91.5 (10.5)	0.014	<0.001		
OGTT					<0.001		
Fasting glucose (mg/dl)	94.4 (10.3)	94.7 (9.8)	97.4 (10.6)^a,b^	0.009	<0.001		
One-hour glucose (mg/dl)	175.9 (35.3)	172.2 (32.5)	180.0 (33.0)	0.182	<0.001		
Two-hour glucose (mg/dl)	138.6 (32.1)	145.7 (27.3)	147.6 (28.1)^a^	0.028	<0.001		
HbA1c (%)	5.3 (0.4)	5.5 (0.5)^a^	5.6 (0.5)^a^	<0.001	<0.001		
HDL cholesterol (mg/dl)	74.4 (14.6)	64.3 (11.0)^a^	54.1 (11.2)^a.b^	<0.001	<0.001		
Triglycerides (mg/dl)	118.4 (32.6)	175.3 (31.5)^a^	259.0 (70.3)^a,b^	<0.001	<0.001		
TyG index	4.6 (0.1)	4.8 (0.1)^a^	5.0 (0.1)^a.b^	<0.001	<0.001		
TG/HDL ratio	1.6 (0.4)	2.7 (0.3)^a^	5.0 (1.7)^a^	<0.001	<0.001		
Pregnancy and delivery outcomes							
Weight gain during pregnancy (kg)	9.4 (6.0)	9.1 (6.6)	9.6 (5.7)	0.716	0.763		
Insulin use, n (%)	71 (33)	79 (36.6)	78 (36.3)	0.694	0.48	2	0.732
Current obstetric complications^*^, n (%)	18 (8.9)	26 (13.1)	25 (13)	0.33	0.207	2	2.215
Maternal complications^**^, n (%)	88 (41.9)	97 (47.1)	103 (50.7)	0.194	0.072	2	3.277
Preeclampsia, n (%)	3 (1.5)	4 (2.0)	7 (3.6)	0.285	0.115	2	2.078
Pregnancy-induced hypertension, n (%)	3 (1.5)	6 (3.0)	8 (4.1)	0.285	0.115	2	2.51
Cesarean, n (%)	114 (53)	113 (53.3)	138 (64.2)	0.029	0.02	2	7.088
Neonatal outcomes^***^							
Gestational age at delivery (weeks)	38.6 (1.2)	38.4 (1.2)	38.4 (1.1)	0.193	0.261		
Birth weight (kg)	3.2 (0.4)	3.2 (0.5)	3.3 (0.4)	0.107	0.035		
Newborn classification, n (%)				0.085	0.103	4	8.192
Small for gestational age	23 (10.6)	18 (8.4)	14 (6.6)				
Adequate for gestational age	182 (83.9)	170 (79.4)	182 (85.4)				
Large for gestational age	12 (5.5)	26 (12.1)	17 (8)				
Jaundice, n (%)	41 (20)	50 (24.4)	54 (27)	0.246	0.098	2	2.803
Newborn hypoglycemia, n (%)	24 (11.7)	25 (12.2)	20 (10)	0.765	0.59	2	0.534
Neonatal ICU, n (%)	8 (3.9)	5 (2.4)	8 (4)	0.625	0.963	2	0.938
Malformation, n (%)	4 (2.0)	5 (2.5)	6 (3)	0.791	0.495	2	0.468
Neonatal death, n (%)	2 (1.0)	1 (0.5)	0 (0)	—	—	2	1.969

The association between TG/HDL, treated as a continuous variable, and markers of glucose metabolism during pregnancy, as well as the TyG index as a proxy for insulin resistance, was assessed using linear regression analysis and is presented in Table 2. The final model was adjusted for age and pre-gestational BMI. TG/HDL showed an inverse association with first-trimester fasting glucose and a positive association with OGTT fasting glucose in the second/third trimester, HbA1c, and the TyG index, even after adjustments. The association with the OGTT two-hour glucose was borderline significant.

**Table 2:** Association between the TG/HDL ratio during pregnancy and markers of glucose metabolism in women with GDM Linear regression analysis examining associations of the TG/HDL ratio with first-trimester fasting glucose, fasting and two-hour glucose from the OGTT, HbA1c, and the TyG index. The final model was adjusted for age and pre-gestational BMI. TyG index: log [(fasting triglycerides (mg/dL) × fasting glucose (mg/dL)) / 2] HbA1c, glycated hemoglobin; OGTT, oral glucose tolerance test; TG/HDL, triglyceride/high-density lipoprotein cholesterol; TyG, triglyceride-glucose index

Variable	Crude		Final model	
	β (95% CI)	P	β (95% CI)	p
Fasting glucose first trimester	-0.759 (-1.418 to -0.099)	0.024	-0.779 (-1.422 to -0.136)	0.012
Fasting glucose OGTT	0.847 (0.362 to 1.332)	0.001	0.795 (0.305 to 1.285)	0.002
Two-hour glucose OGTT	1.389 (-0.120 to 2.899)	0.071	1.491 (-0.053 to 3.036)	0.058
HbA1c	0.039 (0.016 to 0.063)	0.001	0.038 (0.014 to 0.061)	0.002
TyG	0.099 (0.093 to 0.104)	<0.001	0.099 (0.093 to 0.104)	<0.001

**Figure 1:**
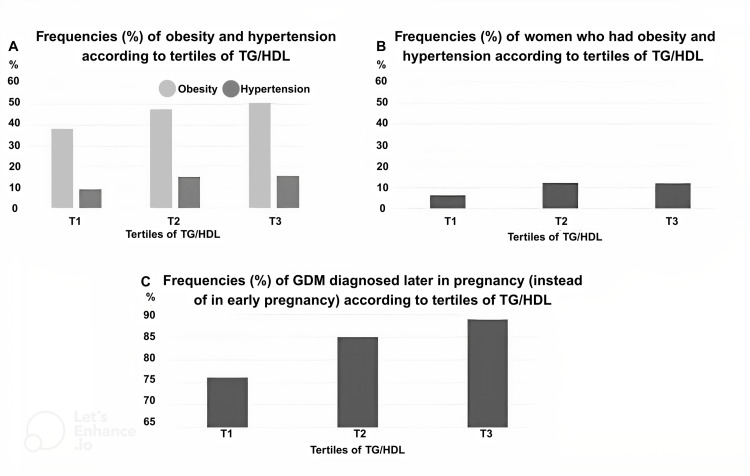
Prevalence of obesity, hypertension, and timing of GDM diagnosis according to TG/HDL tertiles in women with GDM TG/HDL tertiles: tertile 1: <2.22; tertile 2: 2.22-3.37; tertile 3: >3.37 Y-axis: percentage of women with GD. GDM, gestational diabetes mellitus; TG/HDL, triglyceride/high-density lipoprotein cholesterol

Figure 1 illustrates the increasing prevalence of obesity and hypertension across TG/HDL tertiles. In panel A, the frequency of obesity increased significantly from tertile 1 to tertile 3 (p for chi-squared test = 0.005; p for linear trend < 0.001), while hypertension showed a nonsignificant upward trend across tertiles (p for chi-squared test = 0.092; p for linear trend = 0.057). In panel B, the proportion of women with both obesity and hypertension increased significantly from tertile 1 to tertiles 2 and 3 (p for chi-squared test = 0.052; p for linear trend = 0.005). Panel C shows that the prevalence of GDM diagnosed in the second or third trimester rose progressively across TG/HDL tertiles, with statistically significant differences (p for chi-squared test < 0.001; p for linear trend < 0.001).

The association between TG/HDL tertiles and hypertension, obesity, and timing of GDM diagnosis was assessed using logistic regression, as shown in Table 3. The final models for obesity and hypertension were adjusted for age, while the model for GDM diagnosis in the second/third trimester was adjusted for both age and pre-gestational BMI. The TG/HDL ratio was significantly associated with obesity in the third tertile, both before and after adjustment, while the association in the second tertile was borderline significant. For hypertension, associations with the second and third tertiles were marginally significant in both crude and adjusted models. Notably, a strong and significant association was observed between higher TG/HDL tertiles (second and third) and GDM diagnosed in the second or third trimester, which remained significant after adjustments.

**Table 3:** Association of the TG/HDL ratio during pregnancy with hypertension, obesity, and diagnosis of GDM in the second and third trimesters among women with GDM Logistic regression analysis was performed. The final models for obesity and hypertension were adjusted for age. The final model for GDM diagnosis in the second/third trimester was adjusted for age and pre-gestational BMI. TG/HDL tertiles: tertile 1: <2.22; tertile 2: 2.22-3.37; tertile 3: >3.37 GDM, gestational diabetes mellitus; TG/HDL, triglyceride/high-density lipoprotein cholesterol

Variable	Crude						Final model					
	T1		T2		T3		T1		T2		T3	
	OR (95% CI)	P	OR (95% CI)	p	OR (95% CI)	p	OR (95% CI)	p	OR (95% CI)	p	OR (95% CI)	p
Obesity	1	0.028	1.468 (0.994-2.166)	0.053	1.665 (1.128-2.458)	0.01	1	0.032	1.455 (0.986-2.148)	0.059	1.654 (1.120-2.443)	0.011
Hypertension	1	0.109	1.761 (0.960-3.229)	0.068	1.882 (0.993-3.345)	0.053	1	0.113	1.744 (0.951-3.200)	0.072	1.821 (0.992-3.344)	0.053
Diagnostic in the second/third trimester	1	0.001	1.757 (1.083-2.852)	0.022	2.658 (1.560-4.529)	<0.001	1	0.001	1.895 (1.148-3.131)	0.012	2.776 (1.610-4.786)	<0.001

## Discussion

Pathophysiology

Identifying distinct CM profiles among women with GDM is important for guiding preventive strategies aimed at reducing adverse maternal-fetal outcomes both short- and long-term. Our study demonstrated that higher TG/HDL ratios are associated with more adverse CM profiles in pregnant women with GDM. This includes higher rates of obesity and hypertension, poorer glucose and lipid metabolism, and elevated TyG index values, a recognized marker of insulin resistance.

From a pathophysiological standpoint, elevated TG levels serve as an indirect indicator of free fatty acids, reflecting insulin resistance or deficiency. The inverse relationship between TG and HDL, both key players in lipoprotein transport, supports TG/HDL’s value as a surrogate marker for insulin resistance. Our results align with the idea that TG/HDL reflects insulin resistance severity in GDM, similarly to the TyG index, which also progressively increased across TG/HDL tertiles in this study. Previous research has linked higher TyG index values with increased risk of type 2 diabetes and cardiovascular disease [13,14]. Moreover, recent evidence shows that insulin resistance in adipose tissue, independent of hepatic insulin resistance, significantly contributes to elevated triglycerides and reduced HDL-C. This is driven by early defects in adipocyte insulin signaling pathways, including downregulation of insulin receptor, IRS-1, and AKT2, disrupting lipolysis and lipogenesis and impairing plasma lipid homeostasis [15]. Large-scale epidemiological data further confirm that the TG/HDL ratio excels at identifying atherogenic dyslipidemia and insulin resistance, outperforming other indices such as TyG and METS-IR, with area under the curve values approaching 1.0 [16]. These physiological and clinical insights together support the use of TG/HDL as a practical surrogate for insulin resistance in research and clinical practice.

Clinical implications

The main clinical advantage of TG/HDL is its accessibility. Traditional insulin resistance measures like fasting insulin or HOMA-IR are difficult to obtain during pregnancy, but TG/HDL offers a convenient alternative. Our findings showed a direct linear association between TG/HDL and pre-gestational BMI, a parameter often underreported or unavailable in routine clinical settings. Additionally, women in the highest TG/HDL tertile had higher rates of late-onset GDM diagnoses (second/third trimester). This pattern aligns with the physiological increase in insulin resistance induced by placental hormones later in pregnancy [17,18], suggesting that TG/HDL could help identify metabolically at-risk women who might otherwise be missed until later stages.

Despite these associations, maternal-fetal outcomes did not differ significantly between TG/HDL tertiles. This could reflect the success of multidisciplinary glycemic control or limited statistical power to detect subtle or infrequent complications. Nonetheless, the associations between TG/HDL and insulin resistance markers, CM comorbidities, and GDM diagnosis timing highlight its potential clinical relevance.

Comparison with prior studies

Our results are consistent with prior studies linking TG/HDL with components of metabolic syndrome in nonpregnant populations [19], as well as those connecting obesity in pregnancy with adverse outcomes such as preeclampsia, hypertensive disorders, cesarean delivery, and childhood obesity [20-23]. A recent meta-analysis of 33 studies including 23,792 participants found that women with GDM had significantly higher TG/HDL ratios along with elevated LDL and total cholesterol [24]. Other studies reporting increased GDM prevalence in higher TG/HDL tertiles [25,26] may reflect the metabolic burden caused by hormonal changes in late pregnancy [27]. The increased diagnosis of GDM in later trimesters among women with the highest TG/HDL tertile supports the hypothesis that late-onset GDM is more strongly linked to insulin resistance, whereas early-onset GDM may involve more insulin deficiency. This aligns with evidence associating late GDM with more severe complications such as macrosomia and hypertensive disorders [28,29].

Strengths and limitations

This study’s strengths include a large, well-characterized cohort of women with GDM from a specialized tertiary care center, improving internal validity. The use of real-world clinical data enhances translational relevance. Our analyses incorporated both traditional and emerging metabolic markers like the TyG index and used standardized GDM diagnostic criteria. We collected relevant clinical variables such as parity, gestational weight gain, and insulin use, enabling robust subgroup analyses. All lipid measurements were fasting samples, enhancing data quality.

However, limitations must be noted. The cross-sectional design precludes causal inference or temporality. Family history of diabetes was unavailable, limiting adjustment. Statistical issues include the shared triglyceride component in TG/HDL and TyG and no correction for multiple comparisons. Although multicollinearity was not formally assessed (e.g., variance inflation factors), the logarithmic transformation in TyG likely reduced linear correlation with TG/HDL, mitigating multicollinearity risk. Key biomarkers of atherosclerosis or endothelial dysfunction were not measured, and no longitudinal lab quality control data were available. The use of cohort-specific tertiles was exploratory; these cutoffs need external validation before clinical application. Lastly, the single-center design offers consistency but may reduce generalizability and power for rare outcomes.

## Conclusions

This study highlights the relevance of the TG/HDL ratio as a potential marker for identifying unfavorable CM profiles in women with GDM. Higher TG/HDL levels were linked to greater obesity and hypertension prevalence, poorer lipid and glycemic profiles, increased cesarean delivery rates, and elevated insulin resistance as reflected by the TyG index. Additionally, a higher TG/HDL ratio was associated with later GDM diagnosis, typically in the second or third trimester, emphasizing the intensifying role of insulin resistance as pregnancy progresses. These findings suggest that TG/HDL could serve as a useful marker for characterizing CM risk and late-onset GDM, warranting further prospective validation.
